# Higher Intake of Dietary Magnesium Is Inversely Associated With COVID-19 Severity and Symptoms in Hospitalized Patients: A Cross-Sectional Study

**DOI:** 10.3389/fnut.2022.873162

**Published:** 2022-05-12

**Authors:** Saeedeh Nouri-Majd, Armin Ebrahimzadeh, Seyed Mohammad Mousavi, Nikan Zargarzadeh, Mina Eslami, Heitor O. Santos, Mohsen Taghizadeh, Alireza Milajerdi

**Affiliations:** ^1^Department of Community Nutrition, School of Nutritional Sciences and Dietetics, Tehran University of Medical Sciences, Tehran, Iran; ^2^Research Center for Biochemistry and Nutrition in Metabolic Diseases, Institute for Basic Sciences, Kashan University of Medical Sciences, Kashan, Iran; ^3^Obesity and Eating Habits Research Center, Endocrinology and Metabolism Clinical Sciences Institute, Tehran University of Medical Sciences, Tehran, Iran; ^4^School of Medicine, Tehran University of Medical Sciences, Tehran, Iran; ^5^Department of Nutrition, School of Health, Qazvin University of Medical Sciences, Qazvin, Iran; ^6^School of Medicine, Federal University of Uberlandia (UFU), Uberlandia, Brazil

**Keywords:** COVID-19, COVID-19 severity, COVID-19 symptoms, magnesium, magnesium intake

## Abstract

**Background and Aims:**

Magnesium is an anti-inflammatory mineral that plays a role in the innate immune system, and the relaxation of bronchial smooth muscle warrants additional attention in COVID-19. This study examined the association between magnesium intake and COVID-19 severity and related symptoms in hospitalized patients.

**Methods:**

A cross-sectional study was done enrolling 250 COVID-19 patients aged 18 to 65 years. A validated 168-item online food frequency questionnaire (FFQ) was used to assess dietary magnesium intake. COVID-19 Treatment Guidelines were used to determine COVID-19 severity, and symptoms were evaluated using a standard questionnaire. Crude and adjusted analyses were performed (Model 1: age, sex, and energy intake; Model 2: Model 1 + physical activity, supplements, corticosteroids, and antiviral drugs; Model 3: Model 2 + body mass index).

**Results:**

The mean age of participants was 44.1 ± 12.1 years, and 46% of them had severe COVID-19. Patients at the highest tertile of dietary magnesium intake had lower serum levels of inflammatory biomarkers, including CRP (11.8 ± 2.2 vs. 29.5 ± 2.1 mg/L, *p* < 0.001) and ESR (15.8 ± 2.4 vs. 34.7 ± 2.4 mm/hr, *p* < 0.001), than those at the lowest tertile. After controlling for potential confounders, we observed that a higher dietary magnesium intake was associated with a lower odds of severe COVID-19 (OR: 0.32; 95% CI: 0.15–0.70). Also, we found a significant inverse association between dietary magnesium intake and odds of COVID-19 symptoms.

**Conclusion:**

We found that higher intake of dietary magnesium was inversely associated with COVID-19 severity and symptoms.

## Introduction

Coronavirus Disease 2019 (COVID-19) outbreak rapidly became the most serious threat to global health (1, 2). More than 260 million people worldwide were infected in 2021, with over 5 million deaths (3). Prevention and proper treatment for COVID-19 and genetic variants of severe acute respiratory syndrome coronavirus 2 (SARS-CoV-2) remain a concern (4). Along these lines, COVID-19 has imposed a significant financial burden on the global economy and health (5, 6).

Numerous studies have investigated potential interactions between nutrition and immune function (7, 8). Not surprisingly, more emphasis has been placed on the role of vitamins, minerals, and other functional nutrients against infectious-respiratory diseases, including the array of harmful effects of COVID-19 (9, 10). In this light, evoked potentials by micronutrient supplements, such as vitamin D, B12, vitamin C, zinc, and magnesium, have been tested in COVID-19 patients to boost immune function (11–17). However, further investigation is warranted to determine the effects of dietary intake of functional nutrients instead of greater focus on supplementation.

Among a plethora of nutrients thought to be candidates for immune system enhancement, magnesium stands out due to its recognized effects in reducing inflammation and oxidative stress, playing a role in the cytokine storm, lowering blood pressure, and relaxing airway smooth muscles, thus counteracting systemic and respiratory problems (15, 18–20). It is no wonder that recent studies show that hypomagnesemia is a poor prognostic marker of COVID-19 (21, 22). A cohort study of 83 hospitalized patients revealed a link between low serum magnesium levels and an increased risk of COVID-19 symptoms and mortality (21). In a cross-sectional analysis of 60 patients admitted to the intensive care unit with COVID-19 disease discovered that lower serum magnesium levels were associated with more severe disease (22). Another cross-sectional study found that a higher dietary magnesium intake was associated with improved lung function, airway hyperactivity, and wheezing (23). Additionally, a meta-analysis of cross-sectional studies involving 32,918 participants revealed an inverse relationship between dietary magnesium intake and serum CRP levels (24).

Indeed, the relationship between dietary intake of magnesium and COVID-19 must be better examined, however. To the best of our knowledge, there is no study to examine the relationship between dietary magnesium intake and COVID-19 severity and related complications. Therefore, employing a cross-sectional study, we investigated the association between dietary magnesium intake and COVID-19 symptoms and severity in hospitalized patients.

## Methods

A retrospective cross-sectional study was conducted from June to September 2021 at Shahid Beheshti Hospital in Kashan, Iran. The ethics committee of Kashan University of Medical Sciences approved the study protocol with the registration number of IR.KAUMS.MEDNT.REC.1400.048. All participants signed an informed consent form.

### Participants

**Figure 1:**
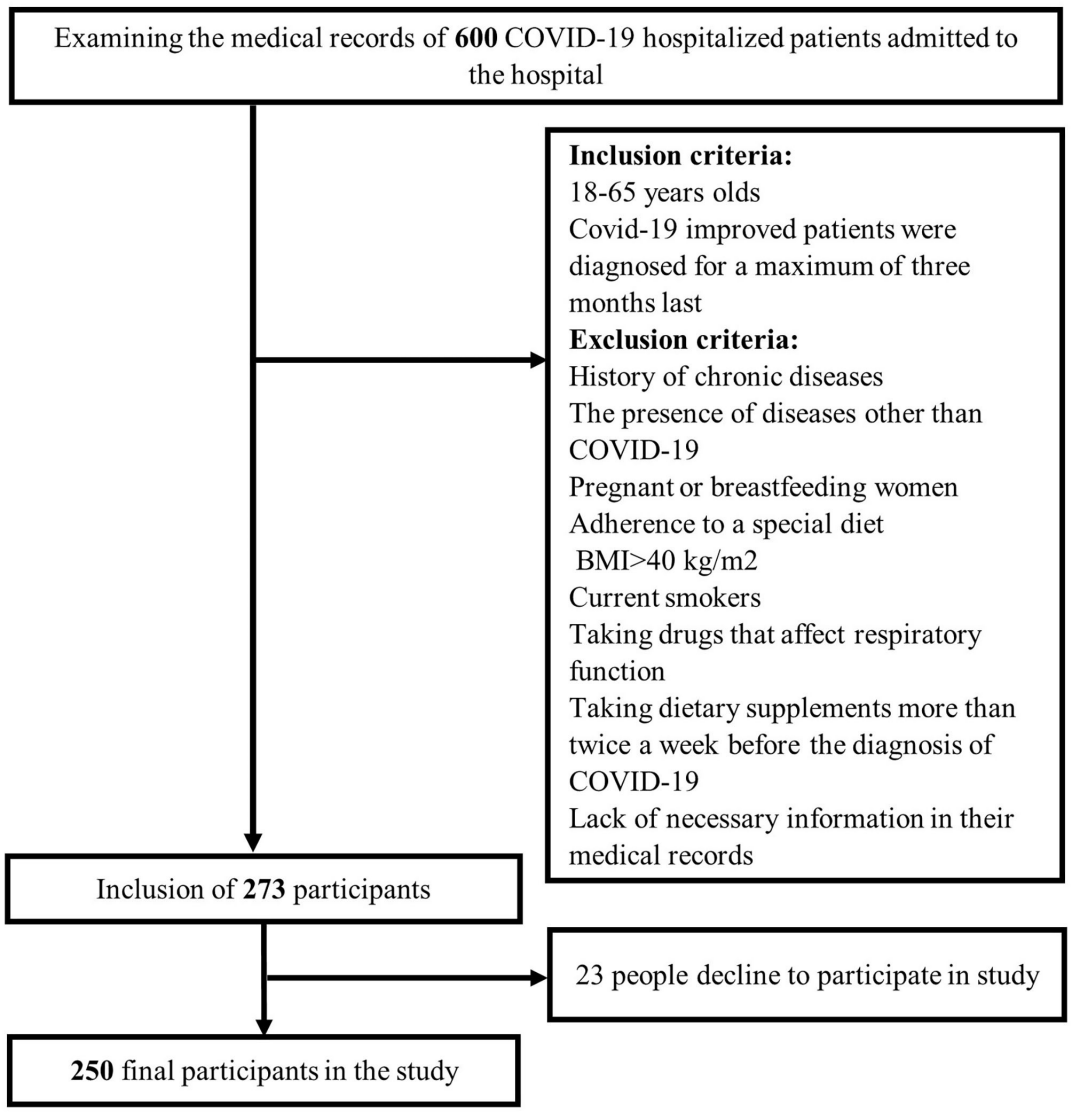
Flowchart of the study's participants.

Using simple random sampling, 250 COVID-19 hospitalized patients aged 18–65 years were included in the study. Participants were selected from improved COVID-19 patients who had been firstly diagnosed for a maximum of 3 months last. Patients who met any of the following criteria were excluded: a history of chronic diseases such as heart disease, diabetes, etc.; the presence of diseases other than COVID-19 as well as diseases that affect the severity of COVID-19; pregnant or breastfeeding women; those who had adherence to a special diet; a body mass index (BMI) >40 kg/m^2^; current smokers; taking drugs that affect respiratory function such as fluticasone, flunisolide, and so on; taking dietary supplements more than twice a week before the diagnosis of COVID-19, as well as a those with lack of necessary information in their medical records (Figure 1).

### Assessment of Dietary Intakes

Patients' dietary information was collected over the past year before being infected by COVID-19 using a 168-item web-based online food frequency questionnaire (FFQ). As the participants in this study were hospitalized and their diets may have changed due to the disease and its complications, we used the FFQ to assess dietary intakes. Participants were able to report their food intake daily, monthly, or annual using this questionnaire. The food consumption was then converted to grams per day using “household measures” (25). Finally, the amounts of dietary micronutrients and macronutrients were determined using the Nutritionist 4 (N4) software.

### Assessment of COVID-19 Severity

COVID-19 Treatment Guidelines (CTG), which were updated on October 19, 2021 (26), were used in this study to determine the severity of COVID-19. According to this tool, the severity of COVID-19 was classified into five levels. (1) Asymptomatic or presymptomatic infection: individuals who had a positive virologic test for SARS-CoV-2 (i.e., a nucleic acid amplification test [NAAT] or an antigen test) but did not exhibit symptoms of COVID-19; (2) Mild illness: individuals who did not have dyspnea, shortness of breath, or abnormal chest imaging but had one or more of the COVID-19 signs or symptoms (e.g., sore throat, weakness, fever, headache, cough, muscle aches, loss of taste and smell, nausea, vomiting, and diarrhea); (3) Moderate illness: individuals who demonstrated evidence of lower respiratory disease during clinical evaluation or imaging and had a room air oxygen saturation (SpO_2_) of 94 % at sea level; (4) Severe illness: individuals with a SpO2 <94% in room air at sea level, a PaO_2_/FiO_2_ ratio <300 mm Hg, a respiratory rate >30 breaths per minute, or lung infiltrates >50%; (5) More severe illness: Individuals who suffered from septic shock, respiratory failure, and/or multiple organ dysfunction. Mild and moderate diseases were considered non-severe diseases in this study.

### Assessment of COVID-19 Symptoms

COVID-19 symptoms were assessed using a standard questionnaire. In this questionnaire, participants were asked to report any of the common COVID-19 symptoms, including fever, chills, cough, sore throat, dyspnea, nausea, vomiting, weakness, and myalgia. These symptoms were confirmed by an infectious disease physician.

### Assessment of Other Variables

A general questionnaire was used to collect data on the participants' demographic characteristics, convalescence duration, physical activity, supplement usage, corticosteroid use, and antiviral medication use, as well as their self-reported weight and height.

### Assessment of Inflammatory Biomarkers

Serum levels of C-reactive protein (CRP) and erythrocyte sedimentation rate (ESR) were measured after the disease diagnosis was extracted from the medical records.

### Statistical Analysis

The Kolmogorov-Smirnov test was used to determine whether data were normally distributed or not. We classified participants into tertiles according to their dietary magnesium intake. The general characteristics of study participants across dietary magnesium intake tertiles were compared using one-way ANOVA and chi-square analysis, respectively, for continuous and categorical variables. After adjusting for age, gender, BMI, and physical activity, we used ANCOVA to compare inflammatory biomarkers (CRP and ESR) between dietary magnesium intake tertiles. Binary logistic regression was used in several models to examine the association between dietary magnesium intake and COVID-19 severity and symptoms. Age (year), sex (male/female), and energy intake (kcal per day) were controlled in the first model. Physical activity (sedentary/moderate/intense), supplementation (yes/no), corticosteroid use (yes/no), and antiviral drug use (yes/no) were additionally controlled in the second model. Finally, BMI was added to the previous model's adjustments. All analyses were conducted using the Statistical Package for Social Sciences (SPSS Inc., version 21). Statistical significance was defined as *P*-values <0.05.

## Results

Table 1 shows the general characteristics of participants across tertiles of dietary magnesium intake. Compared with subjects in the lowest tertile of dietary magnesium intake, patients in the third tertile had a shorter duration of hospitalization (5.9 ± 2.4 vs. 7.2 ± 3.1 days, *p* = 0.007) and convalescence (8.0 ± 2.9 vs. 10.1 ± 3.3 days, *p* < 0.001). Moreover, they were less likely to have overweight or obesity (53.0% vs. 78.3%, *p* = 0.002).

**Table 1:** General characteristics of participants across tertiles of dietary magnesium intake.

	**Tertiles of magnesium intake**			
	**T1 *n = 83***	**T2 *n = 84***	**T3 *n = 83***	*P* ^*^
Age (years)	45.7 ± 11.5	44.2 ± 12.5	42.5 ± 12.3	0.23
Female (%)	59.0	51.2	47.0	0.29
BMI (kg/m^2^)	27.8 ± 3.6	27.8 ± 4.2	25.3 ± 2.7	<0.001
**Physical activity**				0.50
Sedentary	14.5	15.5	7.2	
Moderate	78.3	77.4	86.7	
Intense	7.2	7.1	6.0	
Overweight or obese (%)	78.3	70.2	53.0	0.002
Supplements intake (%)	92.8	95.2	96.4	0.56
Corticosteroids use (%)	91.5	92.3	92.0	0.83
Antiviral Drugs use (%)	91.6	92.9	91.6	0.94
Duration of hospitalization (day)	7.2 ± 3.1	6.5 ± 3.09	5.9 ± 2.4	0.007
Convalescence duration (day)	10.1 ± 3.3	10.3 ± 4.5	8.0 ± 2.9	<0.001

* *Data were obtained from ANOVA or Chi-square test, when appropriate*.

Dietary intakes of study participants across tertiles of dietary magnesium intake are presented in Table 2. Individuals in the top tertile of dietary magnesium intake had higher intakes of energy, carbohydrate, fat, protein, dietary fiber, B vitamins, vitamin C, vitamin D, omega3, calcium, zinc, potassium, and magnesium when compared to those in the lowest tertile. Furthermore, those in the highest tertile of dietary magnesium intake consumed more refined grains, fruits, vegetables, processed meats, fish, poultry, legumes, nuts, high and low-fat dairy. Daily consumption of whole grains and red meat was not significantly different between tertiles of dietary magnesium intake.

**Table 2:** Selected food groups and nutrients intakes of participants across tertiles of dietary magnesium intake.

	**Tertiles of magnesium intake**			
	**T1 *n = 83***	**T2 *n = 84***	**T3 *n = 83***	*P* ^*^
**Nutrients**				
Energy (Kcal/day)	2,554 ± 48.7	2,859 ± 48.4	2,827 ± 48.6	<0.001
Carbohydrate (g/d)	400.1 ± 4.4	412.8 ± 4.2	417.8 ± 4.3	0.01
Fat (g/day)	96.2 ± 2.2	107.8 ± 2.2	98.6 ± 2.2	0.001
Protein (g/day)	95.5 ± 1.1	108.0 ± 1.1	120.7 ± 1.1	<0.001
Dietary fiber (g/day)	19.1 ± 0.3	22.7 ± 0.3	27.7 ± 0.3	<0.001
Vitamin B1 (mg/d)	2.3 ± 0.3	2.5 ± 0.3	2.6 ± 0.3	<0.001
Vitamin B2 (mg/d)	1.7 ± 0.3	1.9 ± 0.3	2.2 ± 0.3	<0.001
Vitamin B3 (mg/d)	26.2 ± 0.3	27.6 ± 0.3	28.5 ± 0.3	<0.001
Vitamin B6 (mg/day)	1.4 ± 0.2	1.8 ± 0.2	1.9 ± 0.2	<0.001
Folate (μg/day)	337.6 ± 6.6	409.4 ± 6.5	501.5 ± 6.5	<0.001
Vitamin B12 (μg/day)	3.3 ± 0.1	4.1 ± 0.1	5.2 ± 0.1	<0.001
Vitamin C (mg/day)	110.7 ± 2.8	134.9 ± 2.7	171.7 ± 2.7	<0.001
Vitamin D (μg/day)	2.4 ± 0.8	2.1 ± 0.7	2.3 ± 0.7	0.01
Omega3 (mg/d)	0.27 ± 0.01	0.42 ± 0.01	0.52 ± 0.01	<0.001
Calcium (mg/day)	832.8 ± 10.1	883.0 ± 9.9	1013.0 ± 9.9	<0.001
Zinc (mg/day)	8.8 ± 0.1	10.4 ± 0.1	11.6 ± 0.1	<0.001
Potassium (mg/d)	3,172.9 ± 36.9	3,730.6 ± 36.0	4,253.6 ± 36.0	<0.001
Magnesium (mg/d)	278.5 ± 2.9	328.9 ± 2.8	379.3 ± 2.8	<0.001
**Food groups (g/day)**				
Refined grains	531.7 ± 16.3	489.4 ± 15.9	490.9 ± 15.9	0.01
Whole grains	72.3 ± 9.1	89.6 ± 8.8	85.1 ± 8.8	0.38
Fruits	278.2 ± 11.0	339.2 ± 10.7	448.7 ± 10.7	<0.001
Vegetables	202.9 ± 8.0	262.7 ± 7.8	367.6 ± 7.8	<0.001
Red meats	41.8 ± 2.2	41.1 ± 2.2	37.7 ± 2.2	0.98
Processed meats	12.5 ± 1.4	15.8 ± 1.4	5.4 ± 1.4	<0.001
Fish	12.5 ± 1.1	21.8 ± 1.1	34.8 ± 1.1	<0.001
Poultry	43.7 ± 2.1	52.1 ± 2.0	68.9 ± 2.0	<0.001
Legumes	103.7 ± 4.0	131.8 ± 3.9	166.7 ± 3.9	<0.001
Nuts	19.8 ± 1.2	33.1 ± 1.2	38.1 ± 1.2	<0.001
Low fat dairy	136.8 ± 7.6	146.2 ± 7.4	184.9 ± 7.3	<0.001
High fat dairy	148.3 ± 7.0	124.5 ± 6.7	126.5 ± 6.7	0.03

*Data are presented as mean ± SE*.

* *All values were adjusted for age, sex and energy intake, except for dietary energy intake, which was only adjusted for age and sex using ANCOVA*.

Table 3 demonstrates the comparison of inflammatory biomarkers across dietary magnesium intake tertiles. Patients in the highest tertile of dietary magnesium intake had lower levels of CRP (11.8 ± 2.2 vs. 29.5 ± 2.1 mg/L, *p* < 0.001) and ESR (15.8 ± 2.4 vs. 34.7 ± 2.4 mm/hr, *p* < 0.001) than those in the lowest tertile. Moreover, patients in the second tertile of dietary magnesium intake had lower levels of CRP (17.7 ± 2.1 vs. 29.5 ± 2.1 mg/L, *p* < 0.001) and ESR (24.3 ± 2.3 vs. 34.7 ± 2.4 mm/hr, *p* < 0.001)compared to those in the lowest tertile.

**Table 3:** Inflammatory biomarkers across tertiles of dietary magnesium intake.

	**Tertiles of magnesium intake**			
	**T1 *n = 83***	**T2 *n = 84***	**T3 *n = 83***	*P* ^*^
CRP (mg/L)	29.5 ± 2.1	17.7 ± 2.1	11.8 ± 2.2	<0.001
ESR (mm/hr)	34.7 ± 2.4	24.3 ± 2.3	15.8 ± 2.4	<0.001

*CRP, C-reactive protein; ESR, erythrocyte sedimentation rate*.

*Data are presented as mean ± SE*.

* *Values were adjusted for age, sex, BMI, and physical activity using ANCOVA*.

The crude and multivariable-adjusted OR with 95% confidence intervals (CI) for severe disease according to tertiles of dietary magnesium intake are presented in Table 4. Higher tertiles of magnesium intake were associated with lower odds of severe illness from COVID-19 in both crude and adjusted models when compared to the lowest tertile. OR for tertile 2 and 3 were, respectively, as follows: 0.45 (0.24–0.83) and 0.24 (0.13–0.47) for crude analysis, 0.39 (0.20–0.76) and 0.21 (0.11–0.43) for model 1, 0.37 (0.18–0.75) and 0.20 (0.09–0.41) for model 2, and 0.41 (0.20–0.85) and 0.32 (0.15–0.70) for model 3.

**Table 4:** Odds ratio (95% CI) of severe disease according to tertiles of dietary magnesium intake.

	**Tertiles of magnesium intake**			
	**T1 *n = 83***	**T2 *n = 84***	**T3 *n = 83***	*P* ^*^
Crude	1	0.45 (0.24–0.83)	0.24 (0.13–0.47)	<0.001
Model 1	1	0.39 (0.20–0.76)	0.21 (0.11–0.43)	<0.001
Model 2	1	0.37 (0.18–0.75)	0.20 (0.09–0.41)	<0.001
Model 3	1	0.41 (0.20–0.85)	0.32 (0.15–0.70)	0.005

*Model 1: Adjustments for age, sex, and energy intake*.

*Model 2: Model 1 + adjustments for physical activity and use of supplements, corticosteroids, and antiviral drugs*.

*Model 3: Model 2 + adjustment for BMI*.

* *Data obtained from Binary logistic regression*.

Crude and multivariable-adjusted OR and 95% CIs for symptoms of COVID-19 according to tertiles of dietary magnesium intake are indicated in Table 5. Higher tertiles of magnesium intake were associated with lower odds of all COVID-19 symptoms assessed (dyspnea, cough, fever, chills, weakness, myalgia, nausea and vomiting, and sore throat) in both crude and adjusted models when compared to the lowest tertile.

**Table 5:** Odds ratio (95% CI) for symptoms of COVID-19 according to tertiles of dietary magnesium intake.

	**Tertiles of magnesium intake**			
	**T1 *n = 83***	**T2 *n = 84***	**T3 *n = 83***	*P* ^*^
**Dyspnea**				
Crude	1	0.51 (0.26–0.99)	0.29 (0.15–0.57)	<0.001
Model 1	1	0.41 (0.19–0.86)	0.23 (0.11–0.48)	<0.001
Model 2	1	0.35 (0.16–0.79)	0.20 (0.09–0.44)	<0.001
Model 3	1	0.38 (0.17–0.87)	0.28 (0.12–0.65)	0.004
**Cough**				
Crude	1	0.28 (0.14–0.54)	0.23 (0.12–0.46)	<0.001
Model 1	1	0.20 (0.09–0.42)	0.16 (0.08–0.35)	<0.001
Model 2	1	0.20 (0.09–0.42)	0.17 (0.08–0.37)	<0.001
Model 3	1	0.20 (0.09–0.45)	0.28 (0.12–0.63)	0.004
**Fever**				
Crude	1	0.24 (0.11–0.56)	0.30 (0.13–0.70)	0.007
Model 1	1	0.20 (0.08–0.48)	0.24 (0.10–0.59)	0.004
Model 2	1	0.19 (0.08–0.48)	0.25 (0.10–0.63)	0.007
Model 3	1	0.21 (0.08–0.52)	0.35 (0.13–0.93)	0.07
**Chills**				
Crude	1	0.21 (0.09–0.50)	0.26 (0.11–0.63)	0.004
Model 1	1	0.18 (0.07–0.45)	0.21 (0.09–0.55)	0.003
Model 2	1	0.17 (0.07–0.44)	0.22 (0.09–0.58)	0.005
Model 3	1	0.19 (0.07–0.48)	0.34 (0.13–0.92)	0.008
**Weakness**				
Crude	1	0.35 (0.18–0.67)	0.13 (0.06–0.28)	<0.001
Model 1	1	0.28 (0.14–0.57)	0.11 (0.05–0.25)	<0.001
Model 2	1	0.27 (0.13–0.56)	0.11 (0.05–0.26)	<0.001
Model 3	1	0.28 (0.14–0.59)	0.14 (0.06–0.34)	<0.001
**Myalgia**				
Crude	1	0.73 (0.40–1.35)	0.36 (0.19–0.68)	0.002
Model 1	1	0.70 (0.36–1.35)	0.35 (0.17–0.69)	0.002
Model 2	1	0.68 (0.35–1.32)	0.33 (0.17–0.67)	0.002
Model 3	1	0.74 (0.37–1.45)	0.45 (0.21–0.94)	0.03
**Nausea and vomiting**				
Crude	1	0.52 (0.24–1.16)	0.04 (0.005–0.29)	<0.001
Model 1	1	0.35 (0.15–0.83)	0.03 (0.003–0.20)	<0.001
Model 2	1	0.36 (0.15–0.86)	0.02 (0.003–0.19)	<0.001
Model 3	1	0.38 (0.16–0.91)	0.03 (0.004–0.24)	<0.001
**Sore throat**				
Crude	1	0.77 (0.41–1.42)	0.09 (0.04–0.23)	<0.001
Model 1	1	0.66 (0.34–1.27)	0.08 (0.03–0.21)	<0.001
Model 2	1	0.66 (0.34–1.28)	0.08 (0.03–0.21)	<0.001
Model 3	1	0.71 (0.36–1.38)	0.10 (0.04–0.29)	<0.001

*Model 1: Adjustments for age, sex, and energy intake*.

*Model 2: Model 1 + adjustments for physical activity and use of supplements, corticosteroids, and antiviral drugs*.

*Model 3: Model 2 + adjustment for BMI*.

* *Data obtained from Binary logistic regression*.

## Discussion

This current cross-sectional study found that higher dietary magnesium intake was associated with lower COVID-19 severity and related symptoms, including dyspnea, cough, fever, chills, weakness, myalgia, nausea and vomiting, and sore throat. More importantly, higher dietary magnesium intake was found to be inversely related to the length of hospitalization and convalescence. Noteworthy, both the second (332 ± 11 mg/d) and the highest tertiles (382 ± 24 mg/d) of magnesium intake were associated with lower odds of severe illness from COVID-19 in both crude and adjusted models when compared to the lowest tertile (273 ± 42 mg/d), but the results were more expressive for the highest one. In this way, the subjects in the highest tertiles of magnesium had a 76, 79, 80, and 68% lowering likelihood of having severe COVID-19 for the crude analysis and adjusted models 1, 2, and 3, respectively, compared to the lowest tertile.

Although no previous studies have examined the relationship between dietary magnesium intake and the symptoms and severity of COVID-19 disease, we are aware that combining vitamin D, vitamin B12, and magnesium was associated with a significant reduction in the need for oxygen support or intensive care in elderly COVID-19 patients (16). Additionally, some studies have been conducted to determine serum magnesium concentrations in COVID-19 patients. For example, a cohort study (*n* = 83) conducted in Wuhan, China, discovered an inverse relationship between serum magnesium levels and COVID-19 symptoms and mortality (21). A cross-sectional study also found lower serum magnesium levels in adults with severe COVID-19 (22). Given that a lower magnesium intake results in lower serum magnesium concentrations (27), we conducted this study to add to the existing body of knowledge. In addition to our research, non-COVID studies support the association between lung function and magnesium intake. A cohort study of children found a relationship between low dietary magnesium intake and poor lung function (28), and a link between increased dietary magnesium intake and improved lung function, airway overreaction, and wheezing in adults was observed through a cross-sectional study (23). Thus, these results provide a biological rationale to our findings related to COVID-19 symptoms, mainly the inherent harmful effects in the respiratory system. Previous clinical trials suggest that magnesium-containing foods can improve the function of the immune system (29, 30). As nutritionally expected, we observed that the highest tertiles for magnesium intake were associated with higher intakes of fruits, vegetables, legumes, and nuts, which are recognized sources of magnesium and display an important role against low-grade inflammation thanks to the food matrix (31). To complement the principal findings of our study, we examined CRP and ESR levels to understand, in part, the inflammatory process, given that severe COVID-19 are associated with higher levels of both biomarkers (32, 33). Regarding CRP values, the difference between tertiles of dietary magnesium intake expresses great clinical magnitude, given that almost three times higher CRP levels were noted in the lowest tertile compared to the highest one (29.5 ± 2.1 vs. 11.8 ± 2.2 mg/L, *p* < 0.001). In agreement with our findings, a 2014 meta-analysis consisting of seven cross-sectional studies (*n* = ~33,000 participants) demonstrated that high magnesium intake was associated with lower serum CRP levels (24). However, this study was conducted prior to the COVID-19 pandemic, and no subsequent study has been conducted so far. Furthermore, another non-COVID-19 study, employing a cohort design, found that magnesium intake was inversely associated with high-sensitivity CRP, interleukin-6, and tumor necrosis factor-alpha concentrations in postmenopausal women (34).

**Figure 2:**
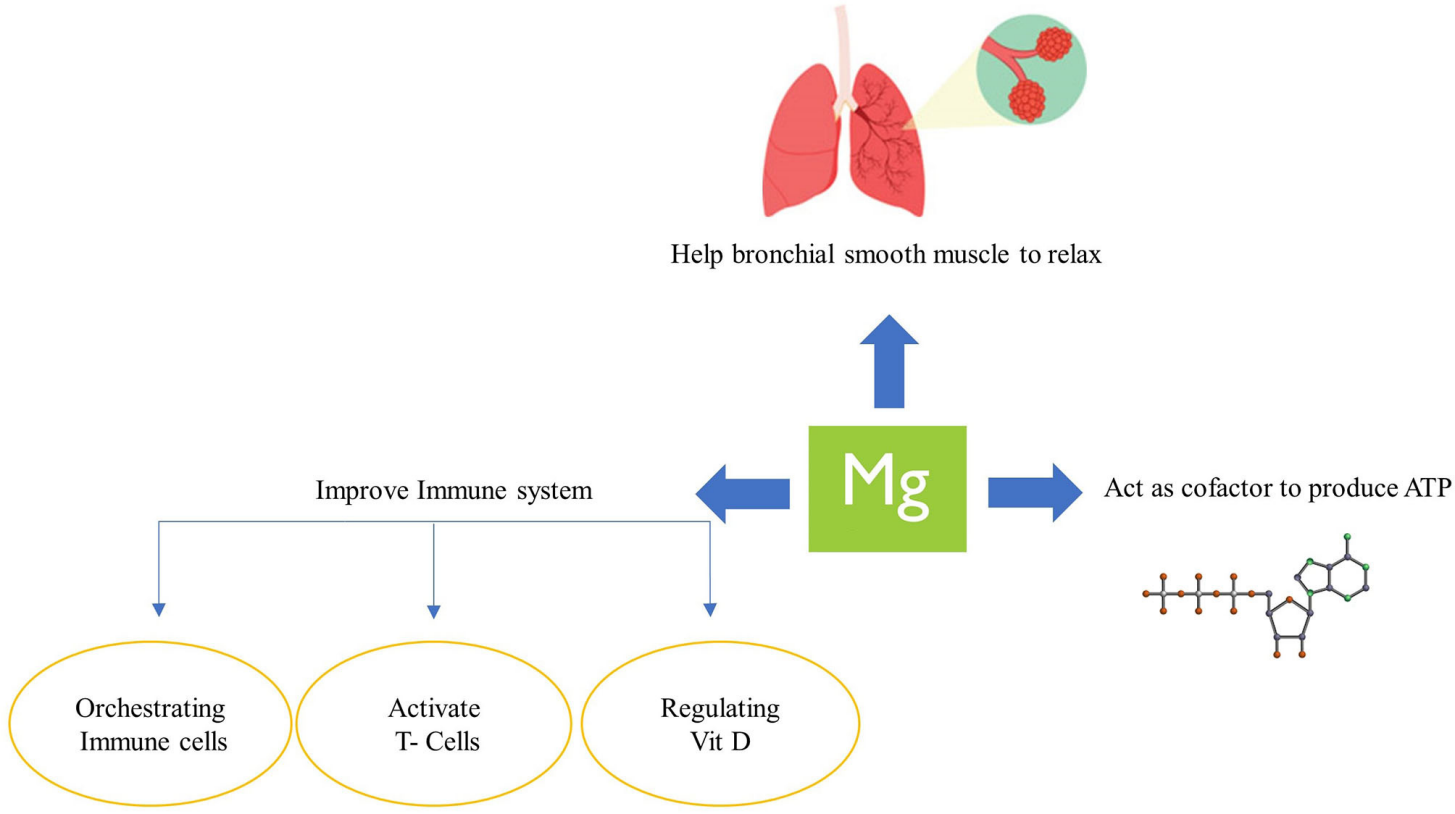
Possible mechanism of the role of magnesium in COVID-19.

COVID-19 can affect multiple organ systems, including the gastrointestinal system, cardiovascular system, liver, kidneys, and respiratory system (20). Cytokine storm is the main cause of organ dysfunctions and death among COVID-19 patients, in which inflammatory cytokines such as interleukins, interferons, chemokines, and tumor necrosis factors are increasingly produced (20). Some mechanisms have been proposed to explain the link between magnesium intake and COVID-19 (Figure 2). Cytokine storm drains ATP in COVID-19, whose regeneration requires magnesium along with phosphate (35). Therefore, adequate magnesium body storage is crucial to avoid an aggressive magnesium deficiency in severe COVID-19 (36). Furthermore, magnesium plays an important role in the relaxation of bronchial smooth muscles, and thus its deficiency might result in respiratory dysfunction in COVID-19 patients (37). The innate immune system is the first line of defense in response to pathogens invasion, such as viral infections like COVID-19 (38). Finally, magnesium regulates the immune system in a variety of ways by orchestrating the activity of immune cells such as neutrophils and macrophages (39), activating T cells via MagT1 (40), and regulating vitamin D activity (41), hence improving the first line of defense in response to pathogens invasion and acting against viral infections like COVID-19 (38).

To the best of our knowledge, this is the first study examining the relationship between dietary magnesium intake and COVID-19 symptoms and severity. In addition to the severity and symptoms of COVID-19 disease, serum levels of inflammatory factors were investigated as predictive indices. Furthermore, many potential confounders were controlled for in this study. However, this study has some limitations that should be taken into account before interpreting the results. First, the cross-sectional design of this study does not allow for inferences about causation to be made. Second, even after adjusting for a variety of potential confounders, the possibility of residual confounding cannot be completely ruled out. Third, this was a single-center study. Although the study population included adults, it would be prudent to consider their sample size and the fact that they were all drawn from the same center when determining their generalizability to the general population. Fourth, despite an infectious disease physician's examination and confirmation of symptoms, we did not use a validated questionnaire to assess COVID-19 symptoms, which may introduce bias into reporting symptoms. Fifth, Using the FFQ to assess study participants' dietary intake could lead to a misclassification of their intake of magnesium. Sixths, serum magnesium levels were not determined to compare with dietary intake results. It would be suggested for future research to consider both the association of dietary magnesium intake and serum magnesium levels. Finally, we did not examine the socioeconomic status of participants, which may influence their dietary intake.

Although the current findings are encouraging, it should be noted that this is prognostic research based on habitual magnesium consumption and thus cannot be extrapolated as part of clinical recommendations. In some severe COVID-19 cases, as well as in other critical care cases, dietary magnesium intake may be insufficient to control serum and general body status; even oral magnesium supplementation may be inadequate in this scenario, and personalized parenteral administration may be required.

## Conclusions

Magnesium is an anti-inflammatory mineral that plays a role in reducing inflammation and oxidative stress during cytokine storms and in relaxing airway smooth muscle. As a result of its role in systemic and respiratory problems in COVID-19, magnesium intake has gained more interest in this regard. While the association between serum magnesium levels and COVID-19 has been studied previously, little attention has been paid to the relationship between dietary magnesium intake as measured by the FFQ and COVID-19 severity and associated symptoms. We found that higher dietary magnesium intake was inversely associated with COVID-19 severity and symptoms in hospitalized patients. More precisely, higher magnesium intake was associated with a shorter duration of hospitalization and convalescence, as well as a lower chance of having COVID-19 symptoms, including dyspnea, cough, fever, chills, weakness, myalgia, nausea, vomiting, and sore throat. Additionally, a higher dietary magnesium intake was associated with lower inflammatory biomarker concentrations (CRP and ESR). We propose that future research examine additional nutrients and minerals that may be associated with COVID-19 severity and related symptoms.

## Data Availability Statement

The raw data supporting the conclusions of this article will be made available by the authors, without undue reservation.

## Ethics Statement

The studies involving human participants were reviewed and approved by Kashan University of Medical Sciences, IR.KAUMS.MEDNT.REC.1400.048. The patients/participants provided their written informed consent to participate in this study.

## Author Contributions

SN-M and SM: conceptualization, formal analysis, writing—original draft, and writing—review and editing. AE, NZ, and ME: data collection. AM and MT: supervision, conceptualization, methodology, investigation, funding acquisition, formal analysis, writing—original draft, and writing—review and editing. HS: writing—review and editing. All authors contributed to the article and approved the submitted version.

## Conflict of Interest

The authors declare that the research was conducted in the absence of any commercial or financial relationships that could be construed as a potential conflict of interest.

## Publisher's Note

All claims expressed in this article are solely those of the authors and do not necessarily represent those of their affiliated organizations, or those of the publisher, the editors and the reviewers. Any product that may be evaluated in this article, or claim that may be made by its manufacturer, is not guaranteed or endorsed by the publisher.
